# Decrease in sexual risk behaviours after early initiation of antiretroviral therapy: a 24-month prospective study in Côte d'Ivoire

**DOI:** 10.7448/IAS.17.1.18977

**Published:** 2014-06-30

**Authors:** Kévin Jean, Delphine Gabillard, Raoul Moh, Christine Danel, Annabel Desgrées-du-Loû, Jean-Baptiste N'takpe, Jérôme Le Carrou, Anani Badjé, Serge Eholié, France Lert, Xavier Anglaret, Rosemary Dray-Spira

**Affiliations:** 1 Epidemiology of Occupational and Social Determinants of Health – Center for Research in Epidemiology and Population Health, INSERM U1018, Villejuif, France; 2 UMRS 1018, Université Versailles Saint-Quentin, Villejuif, France;; 3 PAC-CI Program, CHU de Treichville, Abidjan, Côte d'Ivoire; 4 INSERM U897, Université Bordeaux Segalen, Bordeaux, France; 5 CEPED (Population and Development Research Center – UMR 196 – Paris Descartes/INED/IRD), IRD (Institut de Recherche pour le Développement), Paris, France; 6 Service des Maladies Infectieuses et Tropicales, CHU de Treichville, Abidjan, Côte d'Ivoire

**Keywords:** HIV, AIDS, antiretroviral treatment, sexual behaviours, early ART initiation, HIV prevention, sub-Saharan Africa

## Abstract

**Introduction:**

Whether early antiretroviral therapy (ART) initiation could impact sexual risk behaviours remains to be documented. We aimed to investigate changes in sexual behaviours within the 24 months following an early versus standard ART initiation in HIV-positive adults with high CD4 counts.

**Methods:**

We used data from a prospective behavioural study nested in a randomized controlled trial of early ART (Temprano-ANRS12136). Time trends in sexual behaviours from enrolment in the trial (M0) to 12-month (M12) and 24-month (M24) visits were measured and compared, using Generalized Estimating Equations models, between participants randomly assigned either to initiate ART immediately (early ART) or to defer ART initiation until on-going WHO starting criteria are met (standard ART). Indicators of sexual behaviours included 1) sexual activity in the past year, 2) multiple partnership in the past year, 3) unprotected sex at last intercourse and 4) risky sex (i.e. unprotected sex with a partner of HIV negative/unknown status) at last intercourse.

**Results:**

Analyses included 1952 participants (975 with early ART and 977 with standard ART; overall median baseline CD4 count: 469/mm^3^). Among participants with early ART, significant decreases were found between M0 and M24 in sexual activity (Odds Ratio [OR] 0.72, 95% Confidence Interval [95% CI] 0.57–0.92), multiple partnership (OR 0.57, 95% CI 0.41–0.79), unprotected sex (OR 0.59, 95% CI 0.47–0.75) and risky sex (OR 0.58, 95% CI 0.45–0.76). Among participants with standard ART, sexual behaviours showed similar trends over time. These decreases mostly occurred within the 12 months following enrolment in the trial in both groups and prior to ART initiation in participants with standard ART. For unprotected sex and risky sex, decreases were or tended to be more pronounced among patients reporting that their last sexual partner was non-cohabiting.

**Conclusions:**

In these sub-Saharan adults with high CD4 counts, entry into HIV care, rather than ART initiation, resulted in decreased sexual activity and risky sexual behaviours. We did not observe any evidence of a risk compensation phenomenon associated with early ART initiation. These results illustrate the potential behavioural preventive effect of early entry into care, which goes hand in hand with early ART initiation.

## Introduction

With the preventive effect of early antiretroviral therapy (ART), demonstrated by the HPTN052 trial among stable serodiscordant couples [1], the *Test and Treat* prevention strategy appears as a promising way to curb the HIV epidemic in sub-Saharan Africa [2]. This strategy consists of universal HIV testing, coupled with immediate ART initiation in those diagnosed HIV positive, regardless of their CD4 count. Estimates of the preventive population-level impact of this strategy are mostly derived from models relying on the hypothesis, yet to be proven, that sexual behaviours would not change after early ART initiation [2, 3].

The possibility of risk compensation – increase in risk behaviours as a consequence of decreased perceived risks of HIV burden and/or transmission – may be of particular concern [4, 5]. Increase in sexual risk behaviours associated with ART initiation has been previously reported among high-risk groups early in the ART era [6, 7], and early models predicted that increases in risk behaviours associated with expanded ART could offset the preventive beneficial impact of ART [8, 9]. More recently, risk compensation has been suggested to explain the limited impact of ART for reducing HIV incidence in high-resource settings with high rates of HIV testing and treatment coverage [10]. However, such an effect of ART on sexual behaviours, if any, may vary depending on the context. According to a recent review of 17 observational studies conducted in resource-limited settings [11], only one study conducted in Côte d'Ivoire reported increased unprotected sex after ART initiation [12]. The remaining 16 studies documented decreased levels of sexual risk behaviours associated with ART initiation according to national or international guidelines. These results suggested a beneficial behavioural impact of treatment initiation. They did not investigate, though, whether this effect was due to ART itself or to entry into care.

To date, the consequences of ART initiation on sexual behaviours have mostly been studied in the context of standard ART initiation, that is, among patients with a clinically and/or biologically advanced HIV disease requiring treatment initiation as recommended by the World Health Organisation (WHO) [13, 14]. Health status plays a central role in sexual behaviours, especially in the context of HIV infection [15–17]. Therefore, the effect of ART on sexual behaviours could be different when ART is started earlier, that is, in healthier patients potentially more sexually active. In addition, sexual and preventive behaviours, such as condom use or HIV status disclosure to partners, have been documented to differ according to the type of partnership [18–21], suggesting that ART initiation may have different consequences on sexual behaviours in the case of stable or occasional partnership.

We used data from the on-going Temprano ANRS-12136 randomized controlled trial to measure changes in sexual behaviours within the 24 months following early ART initiation and to compare these changes to those observed in patients starting ART according to WHO guidelines. We also investigated differences in sexual behaviours time trends according to the type of sexual partnership.

## Material & Methods

### Temprano ANRS-12136 trial

Temprano is a multicentre, randomized open-label superiority trial to assess the benefits and risks of initiating ART earlier than currently recommended by WHO, concomitantly or not with a six-month isoniazide prophylaxis for tuberculosis (IPT). The trial was launched in March 2008 in Abidjan, Côte d'Ivoire, and is still on-going. It will end in December 2014. The trial protocol was approved by the Côte d'Ivoire national ethics committee and by the institutional review board of the French National Agency for Research on AIDS and viral hepatitis (ANRS, Paris, France). It has been registered on clinicaltrials.gov under the following identifier: NCT00495651.

Between March 2008 and July 2012, patients attending nine healthcare settings were included in the trial whenever they met the following criteria: informed consent signed; age >18 years; HIV-1-positive; no on-going active tuberculosis; no on-going pregnancy or breastfeeding; CD4 count <800/mm^3^ and no criteria for starting ART according to the most recent WHO guidelines. Participants were randomized into four arms: two “standard ART” arms (arms 1 and 2), in which ART was deferred until patients meet on-going WHO starting criteria [13, 14]; and two “early ART” arms (arms 3 and 4), in which ART was initiated immediately at enrolment. In arms 2 and 4, participants received a six-month IPT, starting at month-1 visit. Once included, participants were asked to show up for trial scheduled visits at day 8, month 1, month 2, month 3, and every three months thereafter. Standardized questionnaires were used to record baseline and follow-up characteristics. The trial sample included 2076 participants. Each participant will be followed-up during 30 months. The main outcome of the trial is the occurrence of a new episode of severe morbidity and any event leading to death.

### Socio-behavioural study

The present socio-behavioural study was nested in the Temprano trial. Starting from 1st January 2010, standardized questionnaires were used to collect information on participants’ sexual behaviours during the past year and on the characteristics of their last sexual intercourse (type of partnership [cohabiting or not]; HIV status of the partner [negative, positive or unknown]; condom use). Questionnaires were administered face-to-face by trained interviewers at enrolment and at 12-month and 24-month visits except for delayed ART initiators in the standard ART arms who completed the questionnaire at enrolment, at ART initiation and then 12 months and 24 months after ART initiation. Patients included before January 2010, although they did not complete a socio-behavioural questionnaire at baseline, participated in the socio-behavioural study during their follow-up at the same tempo as those enrolled from 1st January 2010.

### Study outcomes

Four indicators of sexual behaviours were considered: 1) sexual activity (i.e. at least one sexual intercourse) in the past year; 2) multiple partnership (i.e. at least two sexual partners) in the past year; 3) unprotected sex at last intercourse in the past year; and 4) risky sex (defined as unprotected sex with a partner of HIV negative/unknown status) at last intercourse in the past year.

### Statistical analysis

All trial participants having completed a socio-behavioural questionnaire at one or more of the following trial visits were included in the present analysis: 1) M0 (inclusion visit), 2) M12 (12±3 months after inclusion) and 3) M24 (24±6 months after inclusion). For all analyses, participants of arms 1 and 2 were grouped together in a single group referred to as “standard ART” and participants of arms 3 and 4 were grouped together in a single group referred to as “early ART.”

Time trends in the four indicators of sexual behaviours from M0 to M12 and M24 visits were measured and compared between participants with early versus standard ART. To account for multiple observations per individual, marginal Generalized Estimating Equations models (GEE) of logistic regression assuming an exchangeable correlation structure were used. Covariates included in the models were ART group and time period, coded as a three-level factor in order to allow non-linear changes across time. An interaction term between ART group and time period was added to each model in order to assess differences in time trends according to ART strategy.

In order to investigate different patterns of sexual behaviours according to the type of partnership, we performed interaction tests to assess whether sexual behaviours trends over time differed between sexually active individuals with cohabiting vs. non-cohabiting partners.

Finally, in order to assess the role on behaviours changes of, respectively, entry into care and ART initiation, we described changes in sexual behaviours before versus after ART initiation among participants of the standard ART group. For this complementary analysis, we used GEE models including time periods coded as a four-level factor: 1) M0 (inclusion visit), 2) TI (at Treatment Initiation, allowing for a varying time period between M0 and TI for each individual), 3) TI_+12_ (12±3 months after TI), and 4) TI_+24_ (24±6 months after TI).

All analyses were conducted using SAS version 9.3 (SAS Institute, Cary, North Carolina, USA).

## Results

### Study population

A total of 1952 participants (standard ART: 977; early ART: 975) completed at least one socio-behavioural questionnaire in due time and were included in the present analysis, accounting for a total of 3364 questionnaires (standard ART: 1653; early ART: 1711). As of March 1st, 2013, participants had been followed during a mean time of 25.7 months (Interquartile Range [IQR]: 23.9–30.0), and 57% of participants had completed at least two socio-behavioural questionnaires, with no difference between both ART groups (Supplementary file).

Median age at baseline was 35 years and 79% of participants were women. Median baseline CD4 cell count was 469/mm^3^ (IQR 379–577). No significant difference in baseline socio-demographic and clinical characteristics was observed between patients of standard vs. early ART groups (Table 1).

**Table 1:** Baseline socio-demographic and clinical characteristics of participants of standard and early antiretroviral therapy (ART) groups

	Standard ART	Early ART	*p*
Sex			0.31
Men	219 (22.4%)	200 (20.5%)	
Women	758 (77.6%)	775 (79.5%)	
Age	35 [30–42]	35 [30–42]	0.69
Educational level			0.61
None	236 (24.2%)	257 (26.4%)	
Primary	281 (28.7%)	276 (28.3%)	
Secondary	327 (33.5%)	324 (33.2%)	
> Secondary	133 (13.6%)	118 (12.1%)	
Personal source of income			0.35
No	238 (25.4%)	256 (27.3%)	
Yes	700 (74.6%)	682 (72.7%)	
Family status			0.57
Single	417 (42.7%)	414 (42.5%)	
Living in union	460 (47.1%)	447 (45.8%)	
Separated/widowed	100 (10.2%)	114 (11.7%)	
HIV-status disclosure to the partner			0.92
No	467 (52.0%)	467 (52.2%)	
Yes	432 (48.0%)	428 (47.8%)	
WHO clinical stage			0.88
1	632 (64.8%)	622 (63.8%)	
2	252 (25.8%)	262 (26.9%)	
3	86 (8.8%)	87 (8.9%)	
4	6 (0.6%)	4 (0.4%)	
CD4 count cell (/mm3)	470 [375–573]	468 [384–580]	0.48

Socio-behavioural study nested in the Temprano Trial (*N*=1952); patients in the standard ART group deferred ART initiation until on-going WHO starting criteria were met, whereas patients in the early ART group initiated ART immediately on inclusion in the trial; counts (%) and Chi-squared *p*-values are presented for categorical measures. Percentages are computed as fractions of non-missing observations. Medians (interquartile ranges) and t-test *p*-values are presented for quantitative measures.

### Sexual behaviours within the 24 months following inclusion

**Figure 1:**
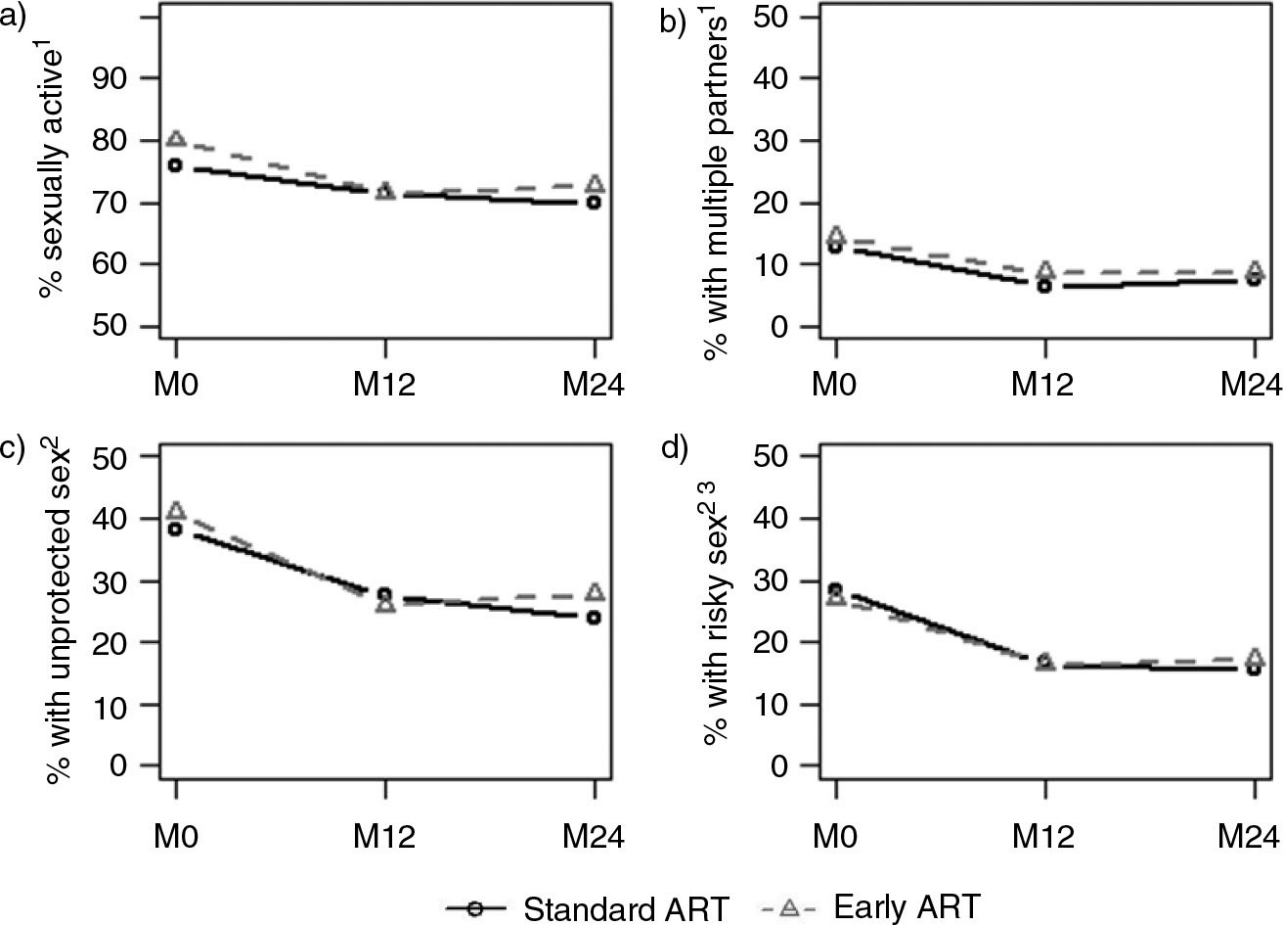
Sexual behaviours reported at inclusion (M0), 12-month visit (M12) and 24-month visit (M24) among participants of standard and early antiretroviral therapy (ART) groups. Socio-behavioural study nested in the Temprano Trial (*N*=1952). Patients in the standard ART group deferred ART initiation until on-going WHO starting criteria were met, whereas patients in the early ART group initiated ART immediately on inclusion in the trial. ^1^In the past year. ^2^At last intercourse in the past year. ^3^Defined as an unprotected intercourse with a partner of negative/unknown HIV status.

The frequency of sexual activity decreased from 79.9% at M0 to 72.6% at M24 among participants with early ART and from 75.9 to 69.8% among participants with standard ART (Figure 1a). At the same time, the frequency of multiple partnership decreased from 14.4 to 8.7% in the early ART group and from 12.8 to 7.6% in the standard ART group (Figure 1b). Frequencies of unprotected sex were 40.7% among participants with early ART and 38.1% among those with standard ART at baseline, decreasing to 27.3 and 23.9%, respectively, at M24 (Figure 1c). The frequency of risky sex decreased from 26.8% at M0 to 17.3% at M24 among participants with early ART and from 28.4% at M0 to 15.5% at M24 among participants with standard ART (Figure 1d).

As shown in Table 2, frequencies of sexual activity, multiple partnership, unprotected sex and risky sex significantly decreased between M0 and M12 in both ART groups (each Odds Ratio [OR] comparing M12 to M0 taking a value of less than 1, with corresponding *p*<0.01); with the exception of sexual activity in the standard ART group, which showed borderline significant decrease over time (OR_M12 vs. M0_ 0.80; 95% Confidence Interval [95%CI] 0.64–1.01]). Subsequently, for the four indicators, the frequencies did not significantly change between M12 and M24 (each *p* >0.05).

**Table 2:** Time trends in sexual behaviour indicators within 24 months following enrolment in the trial among participants of standard and early antiretroviral therapy (ART) groups

	Standard ART				Early ART				
			
*t*_*1*_ *to t*_*2*_	% change^b^	OR (t_2_ vs. t_1_)^c^	95% CI	*p*	% change^b^	OR (t_2_ vs. t_1_)^c^	95% CI	*p*	Interaction *p* a
Sexual activity^d^									**0.61**
** M0 to M24**	−6.1	0.76	[0.59; 0.96]	0.022	−7.3	0.72	[0.57; 0.92]	0.008	
** **M0 to M12	−4.4	0.80	[0.64; 1.01]	0.062	−8.5	0.70	[0.57; 0.87]	0.002	
** **M12 to M24	−1.7	0.94	[0.79; 1.11]	0.45	+1.2	1.03	[0.87; 1.22]	0.77	
Multiple partnership^d^									**0.64**
** M0 to M24**	−5.2	0.55	[0.38; 0.80]	0.002	−5.7	0.57	[0.41; 0.79]	<10^−3^	
** **M0 to M12	−6.4	0.49	[0.34; 0.70]	<10^−3^	−5.8	0.60	[0.44; 0.83]	0.002	
** **M12 to M24	+1.2	1.13	[0.78; 1.64]	0.48	+0.1	0.94	[0.69; 1.27]	0.63	
Unprotected sex^e^									**0.16**
** M0 to M24**	−14.2	0.50	[0.39; 0.64]	<10^−3^	−13.4	0.59	[0.47; 0.75]	<10^−3^	
** **M0 to M12	−10.5	0.61	[0.48; 0.78]	<10^−3^	−14.7	0.55	[0.43; 0.70]	<10^−3^	
** **M12 to M24	−3.7	0.83	[0.68; 1.01]	0.06	+1.3	1.09	[0.89; 1.32]	0.41	
Risky sex^e^,^f^									**0.56**
** M0 to M24**	−12.9	0.48	[0.36; 0.63]	<10^−3^	−9.5	0.58	[0.45; 0.76]	<10^−3^	
** **M0 to M12	−11.8	0.52	[0.39; 0.69]	<10^−3^	−10.4	0.55	[0.42; 0.72]	<10^−3^	
** **M12 to M24	−1.1	0.93	[0.74; 1.17]	0.52	+0.9	1.06	[0.84; 1.34]	0.64	

Socio-behavioural study nested in the Temprano Trial (*N*=1952); patients in the standard ART group deferred ART initiation until on-going WHO starting criteria were met, whereas patients in the early ART group initiated ART immediately on inclusion in the trial

a *p*-value of the overall likelihood-ratio test for interaction between ART group and time (for the whole M0–M24 period)

b change in percentage points between t_1_ and t_2_

c odds ratio of reporting the corresponding sexual behaviour at t_2_ as compared to t_1_ (logistic regression model with Generalized Estimating Equations)

d in the past year

e at last intercourse in the past year

f defined as an unprotected intercourse with a partner of negative/unknown HIV status; M0: at inclusion in the trial; M12: 12 months after inclusion; M24: 24 months after inclusion; OR: Odds Ratio; CI: Confidence Interval.

No significant interaction between study group and time was found for any of the four sexual behaviours indicators (each *p*>0.15), suggesting that time trends between M0 and M24 in these various indicators did not significantly differ across ART strategies.

A complementary analysis was conducted, restricting the GEE analysis 1) to sexually active participants and 2) to participants reporting no condom use at last intercourse. In both cases, the decrease in risky sex between M0 and M12 remained statistically significant (data not shown).

### Differences according to the type of partnership

Among sexually active participants, the overall proportion reporting that their last partner was non-cohabiting was 39.9% at M0; 40.1% at M12; and 42.7% at M24. These proportions were higher among women than men (overall, 44.8% vs. 28.5%, *p*<10^−3^).

Regardless of ART strategy and type of partnership, frequencies of multiple partnership, unprotected sex and risky sex decreased between M0 and M12 (Table 3). For unprotected sex and risky sex, these decreases were or tended to be more pronounced among participants reporting a non-cohabiting partner at last intercourse (OR_M12 vs. M0_ between 0.36 and 0.42) than among those reporting a cohabiting partner (OR_M12 vs. M0_ between 0.60 and 0.77). This differential decrease was not observed for multiple partnerships.

**Table 3:** Time trends in sexual behaviours indicators within 24 months following enrolment in the trial among participants of standard and early antiretroviral therapy (ART) groups, by type of partnership

	Standard ART							Early ART						
	
	Cohabiting partner			Non-cohabiting partner				Cohabiting partner			Non-cohabiting partner			
			
	OR (t_2_ vs. t_1_)^b^	95% CI	*p*	OR (t_2_ vs. t_1_)^b^	95% CI	*p*	Interaction *p* a	OR (t_2_ vs. t_1_)^b^	95% CI	*p*	OR (t_2_ vs. t_1_)^b^	95% CI	*p*	Interaction *p* a
Multiple partnership^c^							0.92							0.48
M0 to M12	0.44	[0.23; 0.84]	0.013	0.52	[0.31; 0.87]	0.012		0.59	[0.36; 0.96]	0.033	0.64	[0.39; 1.05]	0.07	
M12 to M24	1.22	[0.64; 2.32]	0.55	1.16	[0.69; 1.94]	0.57		0.70	[0.39; 1.23]	0.21	0.98	[0.62; 1.54]	0.93	
Unprotected sex^d^							0.038							0.15
M0 to M12	0.77	[0.54; 1.10]	0.15	0.41	[0.26; 0.64]	<.001		0.68	[0.48; 0.95]	0.023	0.41	[0.26; 0.66]	<10^−3^	
M12 to M24	0.68	[0.52; 0.89]	<.001	1.18	[0.79; 1.77]	0.43		1.11	[0.84; 1.46]	0.48	1.10	[0.72; 1.66]	0.67	
Risky sex^d^,^e^							0.27							0.048
M0 to M12	0.60	[0.42; 0.86]	0.006	0.42	[0.26; 0.68]	<.001		0.77	[0.56; 1.07]	0.12	0.36	[0.22; 0.60]	<10^−3^	
M12 to M24	0.78	[0.59; 1.03]	0.08	1.16	[0.76; 1.78]	0.48		0.94	[0.69; 1.28]	0.68	1.33	[0.85; 2.09]	0.21	

Socio-behavioural study nested in the Temprano Trial (*N*=1642 sexually active participants); patients in the standard ART group deferred ART initiation until on-going WHO starting criteria were met, whereas patients in the early ART group initiated ART immediately on inclusion in the trial

a *p*-value of the overall likelihood-ratio test for interaction between type of partnership and time (for the whole M0–M24 period)

b odds ratio of reporting the corresponding sexual behavior at t_2_ as compared to t_1_ (logistic regression model with Generalized Estimating Equations)

c in the past year

d at last intercourse in the past year

e defined as an unprotected intercourse with a partner of negative/unknown HIV status; M0: at inclusion in the trial; M12: 12 months after inclusion; M24: 24 months after inclusion, OR: Odds Ratio: CI: Confidence Interval.

Subsequently, frequencies of multiple partnership, unprotected sex and risky sex generally did not significantly change between M12 and M24, regardless of ART group and type of partnership. The only exception was a significant decrease between M12 and M24 in the frequency of unprotected sex among participant of the standard ART group reporting a cohabiting partner at last intercourse.

### Sexual behaviours before/after ART initiation among participants on standard ART

A total of 802 participants of the standard ART group completed at least one socio-behavioural questionnaire at the following time points: M0, treatment initiation (TI), 12 months after TI (TI_+12_) and 24 months after TI (TI_+24_), representing a total of 1455 questionnaires. Among them, 492 initiated ART (median time between enrolment and treatment initiation: 14.0 months [IQR 8.0–20.1]).

Among these 802 participants, the frequency of sexual activity did not significantly change over time between M0 and TI_+24_ (Table 4). In contrast, frequencies of multiple partnership, unprotected sex and risky sex significantly decreased between M0 and treatment initiation (multiple partnership: OR_TI vs. M0_ 0.41, 95%CI 0.26–0.64; unprotected sex: OR_TI vs. M0_ 0.65, 95%CI 0.49–0.85; risky sex: OR_TI vs. M0_ 0.62, 95%CI 0.45–0.84). Subsequently, the frequencies of these three indicators did not significantly change over time within the 24 months following treatment initiation (each *p*>0.15).

**Table 4:** Time trends in sexual behaviours indicators before and after standard antiretroviral therapy (ART) initiation among participants of the standard ART group

	Sexual activity^a^			Multiple partnership^a^			Unprotected sex^b^			Risky sex^b^,^c^		
			
	OR (t_2_ vs. t_1_)^d^	95% CI	*p*	OR (t_2_ vs. t_1_)^d^	95% CI	*p*	OR (t_2_vs. t_1_)^d^	95% CI	*p*	OR (t_2_vs. t_1_)^d^	95% CI	*p*
M0 to TI	0.91	[0.70; 1.19]	0.50	0.41	[0.26; 0.64]	<10^−3^	0.65	[0.49; 0.85]	0.002	0.62	[0.45; 0.84]	0.002
TI to TI_+12_	0.96	[0.76; 1.21]	0.73	0.98	[0.55; 1.72]	0.93	1.08	[0.84; 1.37]	0.56	0.91	[0.68; 1.21]	0.52
TI_+12_ to TI_+24_	0.96	[0.78; 1.18]	0.68	0.65	[0.34; 1.23]	0.19	0.86	[0.70; 1.09]	0.22	0.88	[0.67; 1.15]	0.35

Socio-behavioural study nested in the Temprano Trial (*N*=802); patients in the standard ART group deferred ART initiation until on-going WHO starting criteria were met

a in the past year

b at last intercourse in the past year

c defined as an unprotected intercourse with a partner of negative/unknown HIV status

d odds Ratio of reporting the corresponding sexual behavior at t_2_ as compared to t_1_ (logistic regression model with Generalized Estimating Equations); M0: at inclusion in the trial; TI: at treatment initiation; TI_+12_:12 months after ART initiation; TI_+24_:24 months after ART initiation; OR: Odds Ratio: CI: Confidence Interval.

## Discussion

In this study nested in an on-going randomized controlled trial of early ART, we found decreases in several reported sexual behaviours within the 24 months following inclusion. These decreases mostly occurred within the first 12 months following enrolment in the trial in both groups and prior to ART initiation in participants with standard ART. They did not differ between participants initiating ART early and those deferring ART according to WHO recommendations, suggesting that such time trends might be a result of early entry into care rather than ART initiation (whether early or not). In addition, regardless of ART strategy, decreases in two sexual risk behaviours indicators, namely unprotected sex and risky sex, tended to be more pronounced for patients reporting non-cohabiting partners as compared to those in cohabiting partnership.

Sexual behaviours in the context of HIV care have been previously investigated in Côte d'Ivoire. Three studies conducted among HIV-positive patients, both treated and untreated, documented levels of sexual activity in the past six months ranging approximately from 50 to 65% [12, 22, 23]. The higher level of sexual activity (71%) during the past year reported in the present study may be explained by a longer recall period. It may additionally be related to the better health status of our study population, made of patients recruited at an early stage of HIV disease. Previous studies also reported levels of unprotected sex, as measured by inconsistent condom use, ranging from 20 to 30% [12, 22, 23]. This is consistent with the 25% participants reporting unprotected sex at last intercourse in our study.

Four indicators of sexual behaviours were used in this study. Among these, risky sex (i.e. unprotected sex with a partner of HIV negative/unknown status) may be considered as the best proxy of partner's exposure to HIV infection. In both ART groups, the odds-ratio of reporting risky sex at last intercourse at M24, as compared to M0, was close to 0.5. This approximately represents, when accounting for the prevalence of risky sex, a 40% decrease at the population level [24]. This indicator integrates different components: sexual activity, condom use and partner's HIV status. Complementary analyses suggested that this time trend reflected not only a decrease in overall sexual activity but also an increase in condom use and in knowledge of partner's HIV status over time. Decrease in sexual activity, number of sexual partners or unprotected intercourses have previously been reported in the context of biomedical prevention trials [25–27]. At the community level, a substantial increase in condom use has also been recently documented in South Africa during ART coverage scale-up [28].

At each time step considered, we found that sexual behaviours did not differ according to ART strategy. This finding might challenge the results of several literature reviews, which pointed out decreased sexual risk behaviours associated with ART initiation [11, 29–31]. However, previous studies relied on comparisons between ART-treated versus untreated patients in a context where routine contacts with the care system are generally infrequent for patients not yet ART-eligible [32]. It has been previously suggested that the behavioural effect of ART may be due to frequent contact with the healthcare system rather than to ART itself, considering that attendance to care provides counselling and psychosocial support [23, 33]. The similar decreases in risk behaviours we found in both ART groups, as well as the absence of additional decrease after treatment initiation in the standard ART group support this hypothesis. Actually, in the Temprano trial the frequency of clinic visits is the same for all participants, regardless of trial arm. Besides, the protocol did not include any additional intervention to reduce risk behaviours apart from routine clinic-based HIV counselling. Our results thus suggest that, as compared to an entry into standard care at early stage of the HIV-infection, early ART initiation per se did not differently impact sexual behaviours.

The dynamics of the changes in sexual behaviours found in this study is consistent with previous results. A study conducted in Uganda indicated a dramatic decrease in unprotected sex at last intercourse during the first year following standard ART initiation, and then a stabilized level during the two subsequent years [34]. Our findings suggest that changes in sexual behaviours occur immediately after inclusion in the trial. These changes might integrate modifications in sexual behaviours resulting from the announcement of HIV diagnosis [35, 36] – which probably occurred shortly before enrolment in our study population with high baseline CD4 levels. Subsequently, after 12 months of follow-up, we did not observe further decrease in sexual behaviours indicators. Neither did we observe any “prevention fatigue” (i.e. a decrease in preventive behaviours over time) as previously reported among high-risk groups [37]. However, our results were obtained within a relatively short follow-up time (24 months). Further studies are needed to measure long term changes in sexual behaviours after early entry into care.

We additionally found that decreases in sexual risk behaviours were more pronounced among patients reporting that their last sexual partner was non-cohabiting versus cohabiting. This differential decrease may reflect a lower level of condom use among cohabiting versus non-cohabiting – or respectively spousal and non-spousal – relationships, as observed in other African settings [19, 20]. It may also reflect the fact that it is more difficult to modify sexual behaviours once they are already established in a couple. In both cases, these results underline the need for specific prevention messages oriented towards well-established couples.

Our results also provide insights into the issue of risk compensation. We did not find any increase in sexual risk behaviours as a result of early ART initiation, an intervention conferring a strong preventive effect against HIV transmission. Actually, we found that sexual risk behaviours were similar whether participants received the intervention or not, and that levels of risk behaviours decreased rather than increased following inclusion in the trial, regardless of ART strategy. These findings, although they provide evidence against the existence of a phenomenon of risk compensation associated with early ART initiation, must be interpreted with caution. Temprano is a clinical trial which primary objective is to measure the individual rather than collective benefits and risks of early ART. Thus, before the implementation of the 2012 WHO guidelines [38], no specific information on the preventive effects of ART was provided to trial participants. Whether participants have received this information from other sources of information following the publication and media exposure of the HPTN052 trial results [1] is unknown.

To our knowledge, this study is the first one to prospectively document detailed sexual behaviours following early ART initiation. Its major strengths are the large and randomized nature of the datasets. However, we acknowledge that our results may be subject to some biases. First, this study relies on self-reported sexual behaviours, which may have been under-reported as a result of social desirability. In order to prevent such a bias, interviewers were trained to administer questionnaires in a non-judgmental way and interviews were conducted confidentially in private rooms. In addition, a literature meta-analysis showed that a face-to-face interview does not always yield to lower estimates of sexual risk behaviours as compared to alternative interviewing tools [39]. Actually, we found higher levels of sexual activity than previous studies conducted among HIV-positive patients in Côte d'Ivoire [12, 22, 23]. This suggests that our results are unlikely to be explained by this sole bias. Second, given the design of the study, it is difficult to disentangle the effect of entry into care from that of enrolment in the trial to explain our results. Of note, Temprano is not a prevention trial but a clinical trial in which only conventional HIV counselling as provided in routine HIV care is offered. Besides, our sample was made of patients recruited in nine clinical centres reflecting the diversity of HIV care offered in Abidjan (hospitals, private clinics, NGO and primary care centres). In each participating centre, all eligible patients were systematically approached to participate in the trial. The total refusal rate was quite low (16%), indicating a limited selection bias. Taken together, these arguments suggest that the trends in sexual behaviours we describe here are also likely to be observed after early entry into care in “real world” settings.

## Conclusions

Through its biological effect, early ART initiation reduces the risk of transmitting HIV from HIV-positive individuals to their sexual partners, which has been documented among the same study population [40]. The present study did not document any evidence of a risk compensation phenomenon associated with early ART initiation. Our results rather suggest that early entry into care, which goes hand in hand with early ART initiation, also carries a substantial behavioural preventive effect. This underlines that, concurrently with the prevention potential of ART, conventional interventions targeting behaviours still have a role to play within combined prevention strategies.
